# Automatic strip layout design in progressive dies using the grouping genetic algorithm

**DOI:** 10.1038/s41598-025-13328-1

**Published:** 2025-08-03

**Authors:** Mehran Afshari, Behrooz Arezoo

**Affiliations:** 1 Department of Mechanical Engineering, Faculty of Engineering, Arak University, Arak, 38156–88349 Iran; 2 CAD/CAPP/CAM Research Center, Department of Mechanical Engineering, Amirkabir University of Technology, Tehran, Iran

**Keywords:** Strip layout design, Grouping genetic algorithm, Punch, Weighted metric method, Engineering, Mechanical engineering

## Abstract

One of the most challenging topics in progressive die design is strip layout design. In the present study, a new method is presented for the automatic strip layout design for progressive dies using the Grouping Genetic Algorithm. A two-objective function is used in the optimization process. The first objective is minimizing the number of stations, and the second is achieving torque equilibrium. The proposed algorithm considers both the minimum number of stations and torque equilibrium simultaneously and is capable of balancing the die torque by adding additional stations (either active or idle) as needed. A software is developed in C# in the Solidworks environment to carry out the algorithm. The inputs to the software are the punch shapes and the constraints between the punches. The output is the strip layout design of sheet metal parts. The performance of the present algorithm is compared with the methods of other researchers and the results indicate that the proposed algorithm performance is improved in the tested cases.

## Introduction

Sheet metal components have been utilized for various industries. Press dies are one of the important tools to produce these components. If the shape of the workpiece is complicated or there are many holes in the part, it is usually produced in several stages. These stages can be implemented with a number of simple dies or with a more complicated progressive die. Using the progressive dies, the cost and time of producing workpieces are decreased and their accuracy is increased. In these dies the workpieces are gradually produced in a number of stations. The progressive die design procedure is complicated and carried out in different steps. Nesting, piloting, punch design, layout design and die components design are some of these steps. These are carried out by experienced designers and need a lot of knowledge. The strip layout design is one of the important and sensitive steps in this industry.

Many studies have been focused on computer-aided die design. Dequan et al.^1^ suggested a knowledge-based system for stamping process planning. Automating strip layout design is considered in their system. In this system, a collision matrix for avoiding the intersection of punches is created and used for strip layout design.

Kumar and Singh developed a number of knowledge-based systems for automating the design of progressive dies. All these systems comprise several Modules that interact with the user through the user interface. These Modules automate various activities in progressive die design. These systems are coded in AutoLISP in the AutoCAD environment. They developed an intelligent knowledge-based system to assist in selecting material for components of progressive die^2^. They also presented another automated system for progressive die modeling^3^. Later they developed an expert system for automating strip layout design in progressive dies^4^. Their system is based on a number of rules. No optimization is utilized in their system. They also proposed a system for automating the piloting of progressive dies^5^.

Ghatrehnaby and Arezoo introduced several systems to automate the design of progressive dies. They proposed an algorithm for automating the nesting and piloting of sheet metal parts^6^. In this system, they proposed a mathematical model for minimizing the scrap in sheet metal part production. They also proposed a system for automating piloting in progressive dies^7,8^. In these systems, a mathematical model is developed using the medial axis transform. They also proposed a mathematical model, using set theory for automating strip layout design in progressive dies^9^. In this study, an algorithm is developed for the layout design by minimizing the number of stations and torque equilibrium. In their system, initially the minimum number of stations is determined. Then punches are assigned in stations to maintain torque balance between the die’s left and right sides. Also, Afshari and Arezoo^10^ proposed a mathematical model for layout design in progressive dies.

Jia et al.^11^ proposed a system for the design of punches and dies in progressive dies of the motor core. Their system comprises three groups of information. Hole-related information, assembly-related constraint attributes and geometry information. Lin et al.^12^ proposed a structural design system for progressive dies in the Catia environment. Their system designs the main components of progressive dies based on predefined databases. Moghaddam et al.^13^ proposed a system for sequence planning of embossing and cutting operations in progressive dies. In their system initially, the operations are classified using three kinds of rules. Then, using fuzzy set theory, the operation groups sequence is determined. Moghaddam et al.^14^ proposed a system to automate nesting and piloting for progressive dies. The system contains three sections. Curve recognition, nesting, and piloting.

Zhang and Spath^15^ proposed a system for estimating the cost of progressive dies used in the manufacturing of electric traction motors, investigating the influence of layout design on die cost. Li et al.^16^ focused on optimizing the accuracy of the trimming line in progressive dies employed for producing complex automotive structural parts. Arora et al.^17^ analyzed progressive dies using SolidWorks software, calculating and evaluating the maximum stress and displacement of die components. Aly et al.^18^ applied a graph-theoretic based method to design and optimize the layout of progressive dies. Shakkarwal et al.^19^ investigated the design of progressive dies using AutoCAD software.

Yang and Hinduja^20^ utilized heuristic rules and fuzzy set theory to design the layout in progressive dies. Murena et al.^21^ developed a web-based system for extracting and identifying features of sheet metal bending processes to enable automated process planning for the production of bending sheet metal parts in progressive dies. Yang et al.^22^ proposed a system for the automatic identification of features in sheet metal parts produced by progressive dies. Skampardonis et al.^23^ employed CAD and analysis software to design and analyze a progressive die capable of performing cutting and forming operations.

Wei et al.^24^ proposed a multilevel modeling strategy to enhance the robustness and automation of stamping die design for automotive panels. Their approach addressed geometric modeling failures by restructuring the design workflow and integrating automated die checking. Faraz et al.^25^ combined Monte Carlo simulation with optimization algorithms to account for material and process variations in bend planning. By integrating probabilistic deflection data with branch-and-bound and TSP-based methods, their approach improved sequence selection and reduced out-of-tolerance results in industrial case studies. Salem et al.^26^ introduced an improved automated system to identify and analyze bending features using STEP AP-203 files. This system interprets the geometry and interrelations of bend lines and introduces a new classification strategy to assist in selecting appropriate tools and sequences.

Prasanth and Shunmugam^27^ proposed a two-stage algorithm for bend sequence optimization in sheet metal process planning. In the first stage, a Bend Feasibility Matrix is used to evaluate manufacturability based on geometric constraints, eliminating infeasible sequences early. The second stage applies a best-first search algorithm to identify a near-optimal bend sequence. Sen et al.^28^ suggested an optimization-based approaches to improve the efficiency and accuracy of bending process planning. They proposed an improved version of the PSO algorithm to avoid local optima.

Jemal et al.^29^ proposed a multi-criteria optimization of press-brake bending for aluminum sheets with various perforation designs. Using finite element simulations in Abaqus, their study examines how different bending angles, sheet thicknesses, and perforation types influence the spring-back effect. Ai et al.^30^ combined intelligent data filtering with classification to recommend suitable process parameters in sheet metal forming. Ghaffarishahri and Rivest^31^ implemented an automated feature recognition method for aerospace sheet metal components. They introduce efficient data structures to organize and analyze STEP models.

Kong et al.^32^ proposed intelligent systems that combine geometric analysis, process knowledge, and optimization techniques to automate stamping layout. The proposed system used feature recognition and combinatorial optimization to arrange trimming, piercing, and flanging operations efficiently to reduce the number of process steps. They^33^ also introduced an intelligent design system that focuses on accurate curve offsetting and surface modeling to handle complex 3D panel shapes.

Xu et al.^34^ used graphical programming to improve the planning of sheet-metal bending operations. In this work, neural networks is applied for predicting inner arc radius based on process parameters. Also, hybrid PSO-GA methods with adaptive inertia weights, is used to optimize multi-step bending sequences. Li et al.^35^ proposed a robust method for recognizing and estimating the pose of irregular sheet metal components in automated production, addressing challenges like detection failures and occlusions. In their work a decoding framework is introduced to identify part types using laser-marked 2D codes, while a contour-based model estimates poses even under occlusion.

Fei et al.^36^ proposed an optimizing process planning for complex sheet metal parts by reducing redundant feasibility checks and improving accuracy. Key methods include reverse state transfer to reuse bending feasibility data and reverse reasoning to limit unnecessary tests. Combination-based experience helps manage solution space growth.

Rathod et al.^37^ developed a system for the automated design of compound dies using Python programming. Jundi and Alaiwi^38^ used mathematical modeling and structural analysis to design a compound die for producing L-shaped products. Ma and Meng^39^ applied size and shape adaptive descriptors to detect anomalies in the production process of stamping progressive dies. Stefanovska and Pepelnjak^40^ utilized the deep neural networks and Light Gradient Boosting Machine to achieve highly accurate predictions of the geometry of sheet metal parts. Molitor et al.^41^ employed machine learning combined with artificial intelligence to detect and address productivity-limiting factors in progressive stamping die.

According to the knowledge of the authors and study of the previous works most of the researchers apply simple rules to carry out the layout design and in most cases optimization is not involved in their operations. Also, no attempt has been reported that simultaneously addresses the two objectives of minimizing workstations and achieving torque equilibrium to solve the layout problem. In the present research, the authors proposed a new approach for automatic strip layout design of progressive dies and tried to address these two deficiencies. A two-objective function namely “torque equilibrium” and “the minimum number of stations” is utilized to solve and optimize the layout problem. To make the balance between these two objectives Weighted Metric Method (WMM) is used for creating the two-objective function. The Grouping Genetic Algorithm (GGA) is utilized for problem optimization. Also, constraint satisfaction between punches is considered. In the present method, the possibility of automatically adding idle or active stations for balancing the die torque is provided.

In this paper, the layout design procedure is explained in Sect."Strip layout design process". The objectives and multi-objective function which are used to optimize the problem are presented in Sect."Objectives for problem solving". In Sect."Constraints between punches"constraints between punches are introduced. The proposed genetic algorithm is put forward in Sect."Proposed genetic algorithm". The software performance is evaluated with several examples in Sect."Examples". The Conclusion is presented in Sect."Conclusion"and finally, references are presented in Sect.“References”.

## Strip layout design process

A number of punches are used to produce each sheet metal part. These punches are placed at different stations of the progressive dies. One of the most challenging steps in progressive die design is the strip layout design process, which strongly affects both part accuracy and die cost. In this process, the number of stations and distribution of punches in these stations are to be determined. This process is complicated and time-consuming and carried out by expert designers.

## Objectives for problem solving

Two objectives are considered to solve the present problem. These objectives are:

- Minimizing the number of stations: Reducing the number of stations reduces the die costs. Therefore, it is considered as the first objective of the problem according to Eq. (1):

1 $$\:\text{Min}\:\:\:\:\:{\text{Z}}_{\text{1}}\text{=m}$$

where *m* is the number of stations.

- Torque equilibrium: Punches should be suitably assigned in an appropriate number of stations so that the torque between the right and left sides of the die is balanced. This is due to the fact that the torque imbalance causes the guide pins to bear high loads and cause asymmetry in the pins and hence increasing the wear rate of the die block and punches. This can also have an effect on the accuracy of final components. The vertical torque (along the Y axis) is constant and does not depend on the arrangement of punches. Therefore only the horizontal moment (along the X axis-along to the direction of motion) should be considered as the objective function. The relation for this objective is according to Eq. (2):

2 $$\:\text{Min}\:\:\:\:{Z}_{2}\text{=}\left|\sum_{i=0}^{n}{F}_{i}\times\:{X}_{i}\right|$$

where $\:{F}_{i}$ is the shear force of the *i*th punch and $\:{X}_{i}$ is the distance of the *i*th punch from the die center.

In problems with several objectives, the possibility of finding results that optimize all objectives is low. So a multi-objective function is used in the present work. In this problem, the WMM which is one of the solving techniques in multi-objective problems, is used. In this method, the distance between a solution and the ideal solution (optimum) is measured^42^. This is shown in Eq. (3):

3 $$\:Z=\sum_{i=1}^{k}{w}_{i}\left|\frac{{Z}_{i}-{Z}_{i}^{min}}{{Z}_{i}^{max}-{Z}_{i}^{min}}\right|$$

In this relationship, $\:{Z}_{i}$ is the value for the *i*th objective function. $\:{Z}_{i}^{min}$ is the minimized value for the *i*th objective function and $\:{Z}_{i}^{max}$ is the maximized value for the *i*th objective function. $\:{w}_{i}$ is the weighting coefficient of the *i*th objective function which is a number between zero and one. Also, the sum of the weights of objective functions is equal to 1 as shown in Eq. (4).

4 $$\:\sum_{i=1}^{k}{w}_{i}=1$$

In the present work two objectives are considered as shown in Eq. (5):

5 $$\:Min\:\:\:\:Z={w}_{1}\left|\frac{{Z}_{1}-{Z}_{1}^{min}}{{Z}_{1}^{max}-{Z}_{1}^{min}}\right|+{w}_{2}\left|\frac{{Z}_{2}-{Z}_{2}^{min}}{{Z}_{2}^{max}-{Z}_{2}^{min}}\right|$$

$\:{w}_{1}$ and $\:{w}_{2}$ are considered to be equal to 0.5.

## Constraints between punches

The punches which are used to produce the workpieces are not arbitrarily assigned to stations. There are some rules and constraints which should be followed to assign punches to the stations. In this section four important constraints are described.

### Intersection constraints

**Fig. 1:**
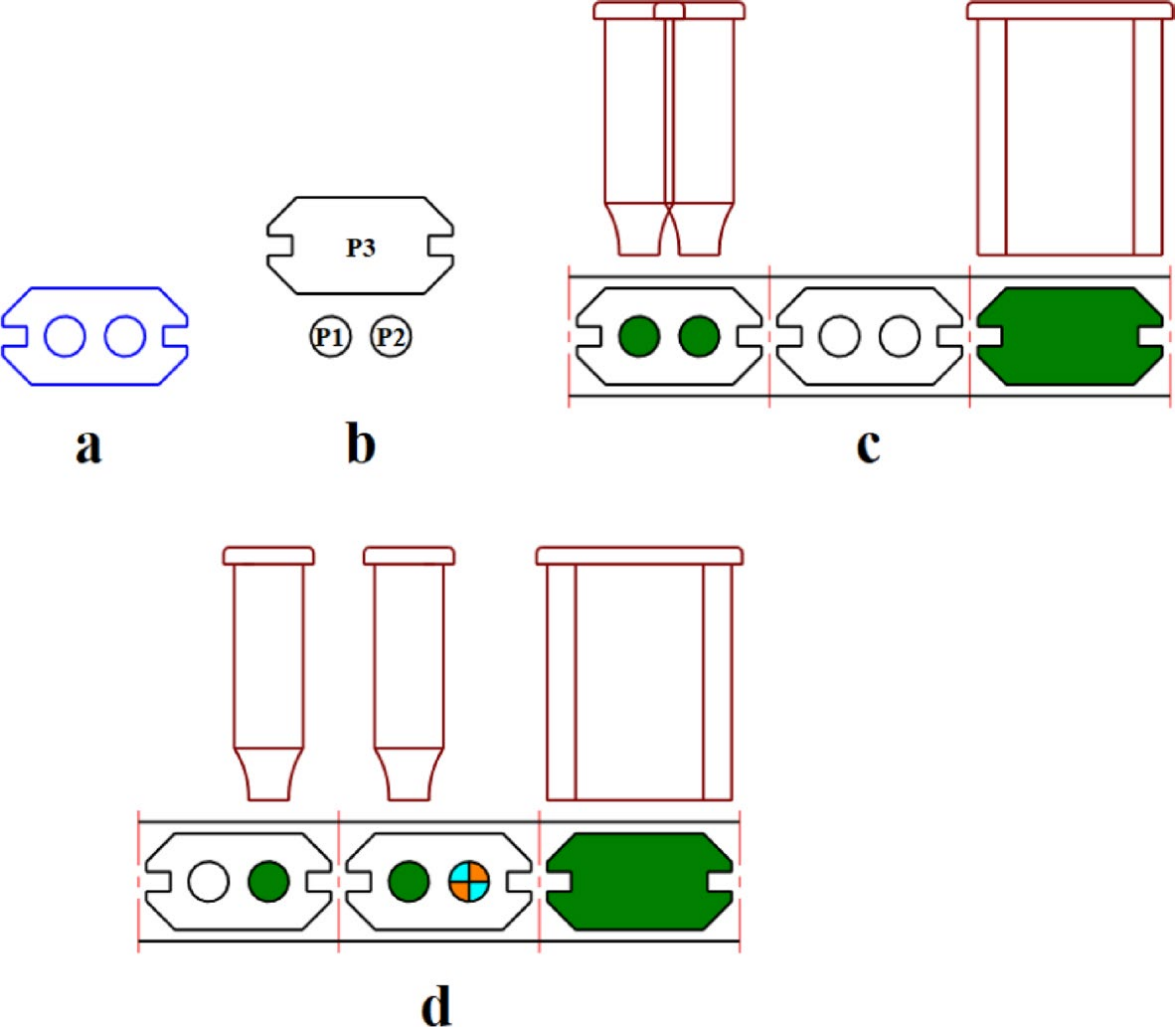
(**a**) Sample sheet metal part, (**b**) Operating punches, (**c**) Intersection shanks of punches with the same operating station, (**d**) Appropriate layout design without the intersection of Punches.

Some punches cannot be allocated to the same station since either they themselves or their shanks may overlap. For example in Fig. 1 punch P3 which is utilized to produce the external contour, overlaps completely with two punches P1 and P2 which are used to produce the holes. Therefore, punch P3 has the intersection constraint with two punches P1 and P2 and cannot be allocated in the same station. In some cases the punches do not intersect with each other but as can be seen in Fig. 1c where punches P1 and P2 are close in a way that their shanks intersect with each other and so cannot be assigned in the same station. These states that two punches cannot be assigned in the same station are considered as intersection constraint.

### Priority constraints

**Fig. 2:**
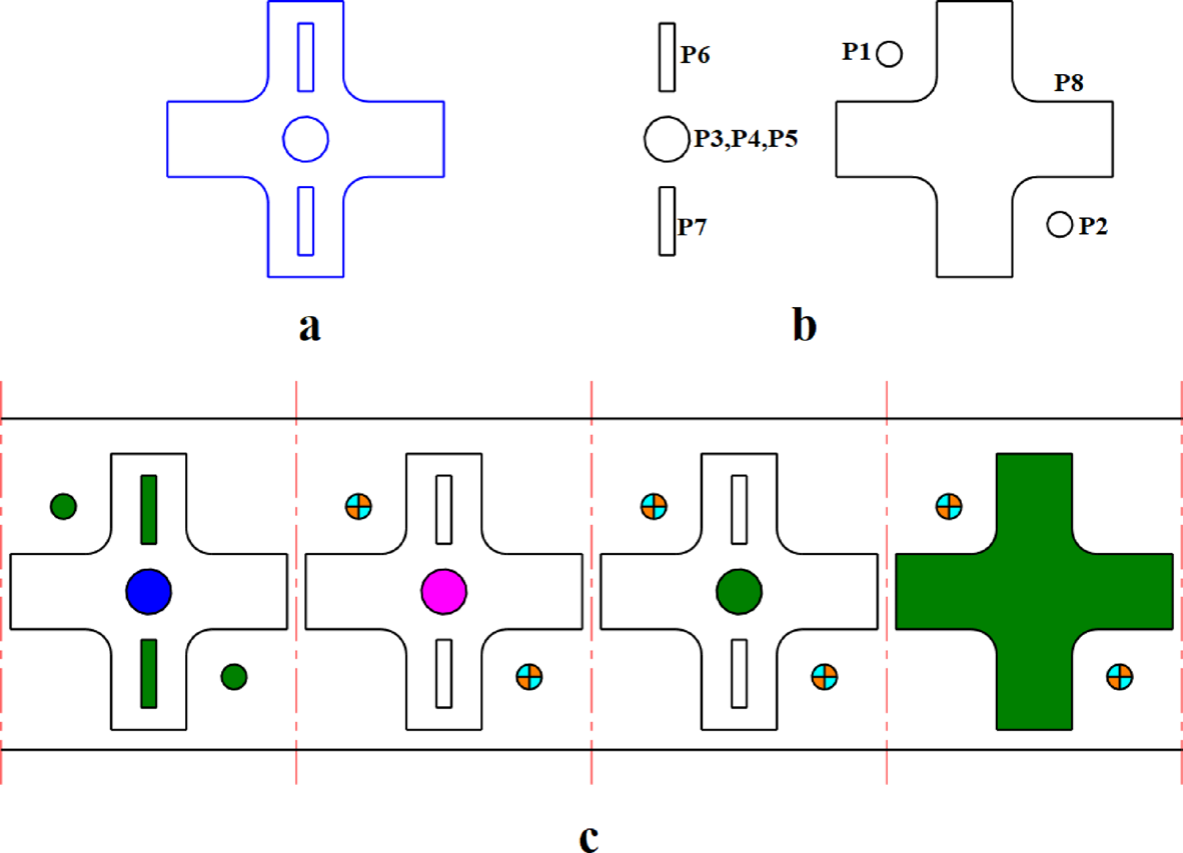
(**a**) Sample sheet metal part, (**b**) Operating punches, (**c**) Strip layout.

In some cases, a punch should operate before one or more punches. For example, some holes are precise without any burrs and have accurate dimensions and geometric tolerances. To produce such holes two or more punches should be utilized. The first punch pierces the sheet metal and the other punch or punches deburr the hole and make it accurate. Therefore the piercing punch should operate before shaving or deburring punches. Also, shaving punches should operate in turn due to their degree of precision. For example, the central hole in Fig. 2a has a tight dimensional tolerance and to produce this hole three punches are used. First, the punch P3 operates to pierce the hole and the punches P4 and P5 operate respectively as shaving punches. Another example is where two holes with large size differences are to be punched in the same station. In this case, the larger hole should be punched first and the smaller hole should be punched in one of the following stations. Otherwise, the smaller hole will be deformed due to material stretch when punching the larger hole.

### Assignment in the first station constraint

In progressive dies, the punches used to create pilot holes should be placed in the first station. For example, in Fig. 2, punches P1 and P2 are used to create pilot holes and are therefore positioned in the first station.

### Assignment in the last station constraint

In progressive dies, the punches used to create external contours or cutting off workpieces should be placed in the last station. For example, in Fig. 2, punch P8 is utilized to create the external contour and is therefore assigned to the last station.

## Proposed genetic algorithm

In the present study, GGA which was introduced by Falkenauer^43^ is used to solve the layout design problem. The steps of this method are described below.

### Problem encoding

**Fig. 3:**
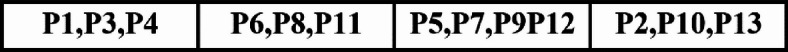
GGA Chromosome used for solving the problem.

GGA encoding is different from the standard Genetic Algorithm (GA). In GGA chromosomes can have different lengths and each chromosome consists of several groups where some objects are assigned to these groups. For the present problem, each chromosome includes a number of stations where some punches are assigned in each station as shown in Fig. 3.

### Initial population

To start the GGA a number of chromosomes are needed as the initial population. The chromosomes are usually generated randomly. In order to create the initial population completely randomly, initially the number of stations should be determined randomly too. Then the punches should be assigned to these stations randomly. Then the constraints between them are checked. If all of the constraints are satisfied, then the produced chromosome is correct and is considered a member of the initial population. Otherwise, the chromosome is excluded from the initial population. Due to various constraints between the punches, producing the initial population with this method is time-consuming. Therefore in the present work, each chromosome is produced according to the following stages:

- The number of stations is determined randomly. This number is between the minimum and the maximum number of stations as Eq. (6). Minimum number of stations is considered as two and the maximum number of stations is considered as the number of punches.

6 $$\:{\text{St}}_{\text{min}} \leq \text{St} \leq {\text{St}}_{\text{max}}$$

- Punches used for piloting are assigned to the first station and punches used for cutting off the workpiece from the strip are assigned to the last station.
- Punches with a priority constraint are randomly added to stations where assignment is possible. If it is not possible to assign a punch, a station is added to the die.
- Punches with an intersection constraint are randomly added to stations that can be placed. If it is not possible to assign a punch, a station is added to the die.
- Punches without constraints are randomly assigned to different stations.

If there are no stations for assigning a punch then a station is added to the existing stations (the position of the added station is determined randomly in places where the punch can be assigned).

The production of a chromosome as a member of the initial population is described with an example as follows. It is assumed that 9 punches are used to produce the workpiece and the constraints between the punches are as shown in Table 1. The five stages to produce the chromosome are explained as follows:

**Table 1:** Constraints between punches for example of production initial population.

Constraint	Punches with this constraint
Intersection	{P1,P6},{P2,P5},{P4,P7},{P4,P5}
Priority	{P3,P5,P7},{P6,P4}
Assignment in the First station	P1
Assignment in the Last station	P9

**Fig. 4:**
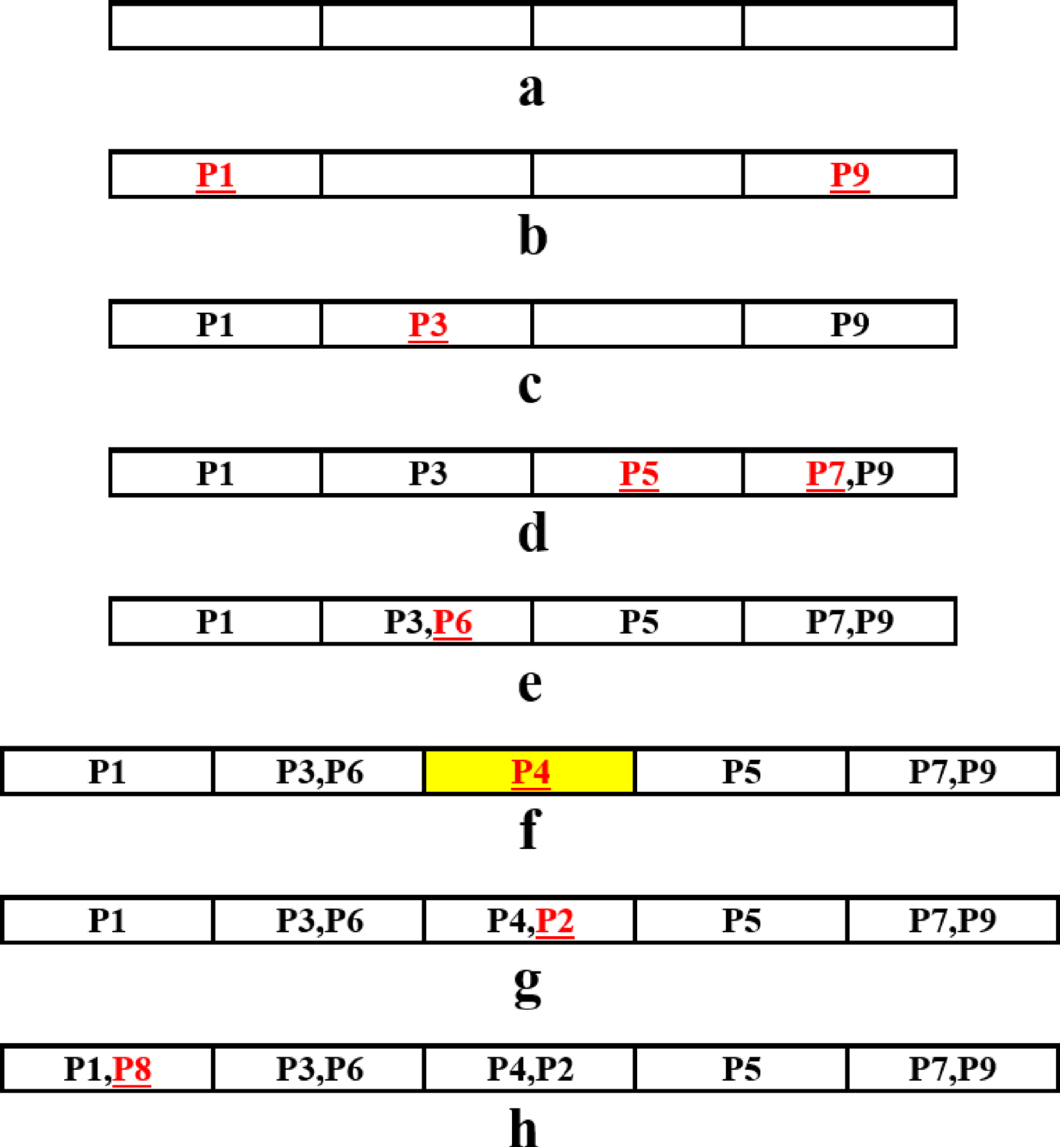
Steps for generating a chromosome. (**a**) Determination of the number of stations, (**b**) Assigning piloting punch and cutting-off punch, (**c**) to (**f**) Assigning punches with priority constraint (**g**) Assigning punches with intersection constraint (**h**) Assigning punches without constraints.

Stage 1: Initially a minimum of 2 and maximum of 9 stations are assumed for strip layout design. Then 4 stations are determined randomly to assign the punches as shown in Fig. 4a.

Stage 2: Punch P1 is used to produce the piloting hole and it is assigned to the first station and punch P9 is used for cutting off the workpiece from the strip and is assigned to the last station (Fig. 4b).

Stage 3: Punches P3, P5, and P7 are categorized within the same group and have priority constraints with each other. The feasible stations for the assignment of punch P3 are limited to stations 1 and 2. This punch cannot be placed in stations 3 and 4 because placing it there would prevent the placement of the other two punches. Between stations 1 and 2, station 2 is randomly selected for punch P3 and is placed there as shown in Fig. 4c. Following this, the only available station for punch P5 is station 3, where it is subsequently placed (Fig. 4d). Similarly, punch P7 is exclusively assigned to station 4 (Fig. 4d).

Punch P6 cannot be assigned at Station 1 due to its intersection with allocated punch P1. Consequently, the feasible stations for assignment of this punch are limited to Stations 2 and 3. Station 2 is randomly selected for its placement (Fig. 4e). Following the assignment of punch P6 to Station 2, the only remaining stations available for punch P4 are Stations 3 and 4. However, punch P4 cannot be placed in these stations either, as it has the intersection constraint with allocated punches P5 and P7. As a result, no feasible station remains for its placement. To resolve this problem, an additional station should be introduced, and punch P4 is positioned there. The possible locations for this new station are between Stations 2 and 3 or between Stations 3 and 4. By random selection, the new station is inserted between Stations 2 and 3, and punch P4 is assigned to this newly added station (Fig. 4f).

Stage 4: Punches P1, P2, P4, P5 and P7 have intersection constraints; however, all of them except punch P2 have already been assigned to different stations during previous stages. Punch P2 has the intersection constraint with punch P5 and, therefore, cannot be placed in Station 4. Consequently, it is randomly assigned to Station 3 (Fig. 4g).

Stage 5: Among the introduced punches, only punch P8 has no constraints and can be placed in all stations. It is randomly assigned in Station 1 (Fig. 4h).

### Crossover

After selecting two parents, the following steps are carried out for crossover:

- Two crossing points are selected for each of the parents.
- The elements of the crossing section of the second parent are inserted at the left crossing point of the first parent.
- Punches that are repeated more than once in stations are determined and their related stations are removed from the first parent. In this state, some punches will remain without any attached stations.
- The constraints between punches are checked and if necessary their stations are corrected. Also, punches without assigned stations should be placed accordingly.
- Steps 2 to 4 are repeated for the second parent.

When the crossover operation is carried out, the priority constraints may be violated. Also, piloting punches and cutting off punches may be replaced from the first or last station. So according to step 4, the constraints between punches should be checked and if necessary the stations of the punches are replaced. Also, punches that are not assigned to any stations should be allocated to one station. Hence, the following steps are utilized to correct the punches location:

- If piloting punches are not assigned to the first station or cutting off punches are not assigned to the last station then their locations are replaced and corrected. Also, if they are without any stations then they are assigned to first and last stations.
- Punch sets with priority constraints attached, are checked from largest to smallest sets. When considering a punch set, initially all punches from this set that are assigned to one of the stations are identified. If these identified punches are not ordered according to their priority order then they are again assigned in the ordered manner.
- Punches without stations attached are assigned to stations as described in the initial population (Sect. "Initial population").

The procedure of crossover is described with an example as follows; it is assumed that fourteen punches are used to produce a workpiece and the constraints between them are shown in Table 2. It is also assumed that punch P1 is the piloting punch and punch P14 is used to cut-off the workpiece from the strip.

**Table 2:** Constraints between punches for the crossover example.

Constraint	Punches with this constraint
Intersection	{P1,P6,P12}, {P6,P2}, {P7,P14}
Priority	{P4,P6,P5,P10}
Assignment in the First station	P1
Assignment in the Last station	P14

**Fig. 5:**
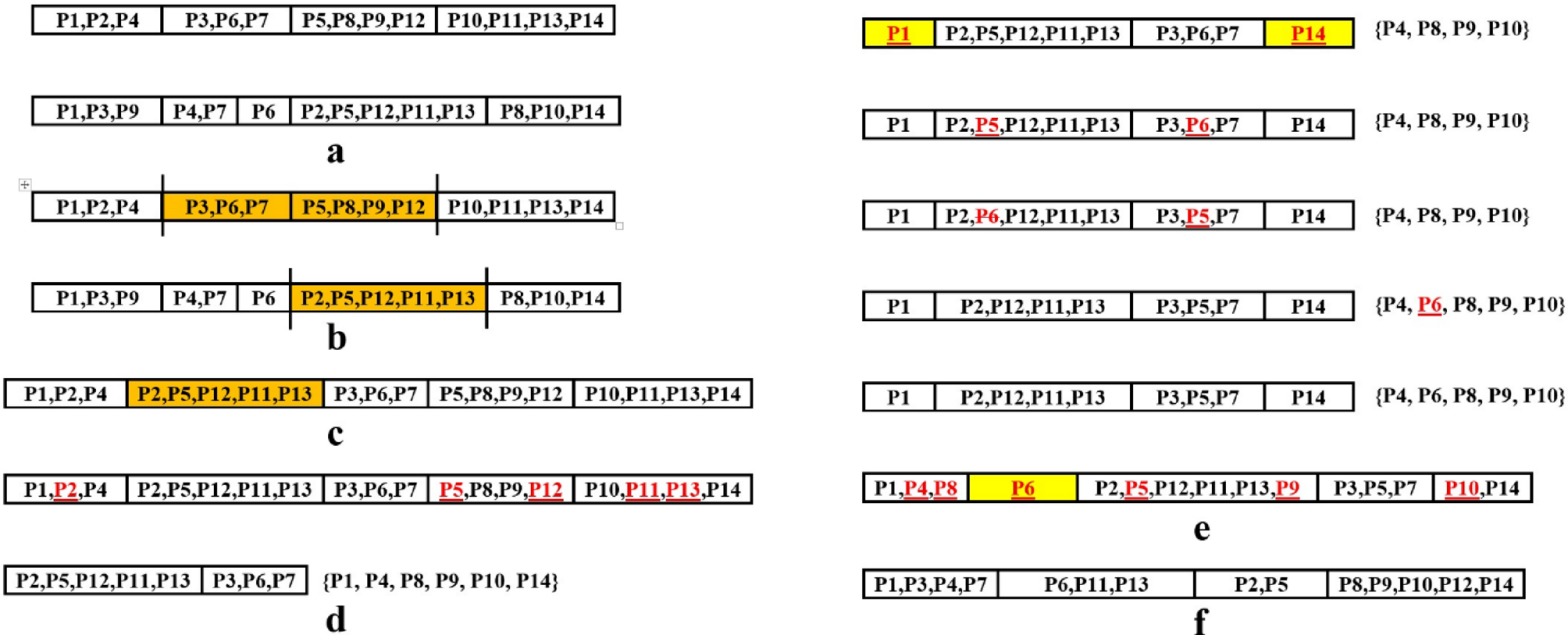
Steps for crossover example. (**a**) Parents, (**b**) Selecting the place of crossover, (**c**) Inserting the crossing section of the second parent at the left side of the crossing point of the first parent, (**d**) Deleting stations of repeated punches from the first parent, (**e**) Checking and correcting constraints between punches and assigning punches without station, (**f**) Second child.

It is assumed that the two selected parents are as Fig. 5a. In the first step the points of the crossover are selected randomly as shown in Fig. 5b. Then elements of the crossing section of the second parent are inserted at the left crossing point of the first parent (Fig. 5c). In next step, punches which are repeated more than once are identified and their related stations removed from first parent (Fig. 5d). In the last step, constraints between punches are checked and the necessary correction is carried out and punches without any stations attached, are assigned (Fig. 5e). As shown in this Fig, initially punches P1 and P14 with predetermined stations are assigned. Due to the intersection of punches P1 and P12, a station is added at the beginning of the stations and punch P1 is assigned to this station. Also, because of the intersection of punch P14 with punch P7, a station is added at the end of the stations and punch P14 is assigned to this station. Then the priority constraint is checked. There is only one punch set with this constraint and it is considered. First, identification, sorting and replacement of punches of this punch set are carried out. Hence, punches P5 and P6 are identified and sorted (P6 before P5) and Punch P5 is assigned to station 3. But P6 has the intersection constraint with punches P2 and P12 and cannot be assigned to station 2 so is put aside. Now punches P3, P4, P6, P8, P9 and P10 have no stations attached and so are assigned to stations according to initial population Sect. 5.2. Also, the second child which is obtained in the crossover operation is produced with similar steps (Fig. 5f).

### Mutation

**Fig. 6:**
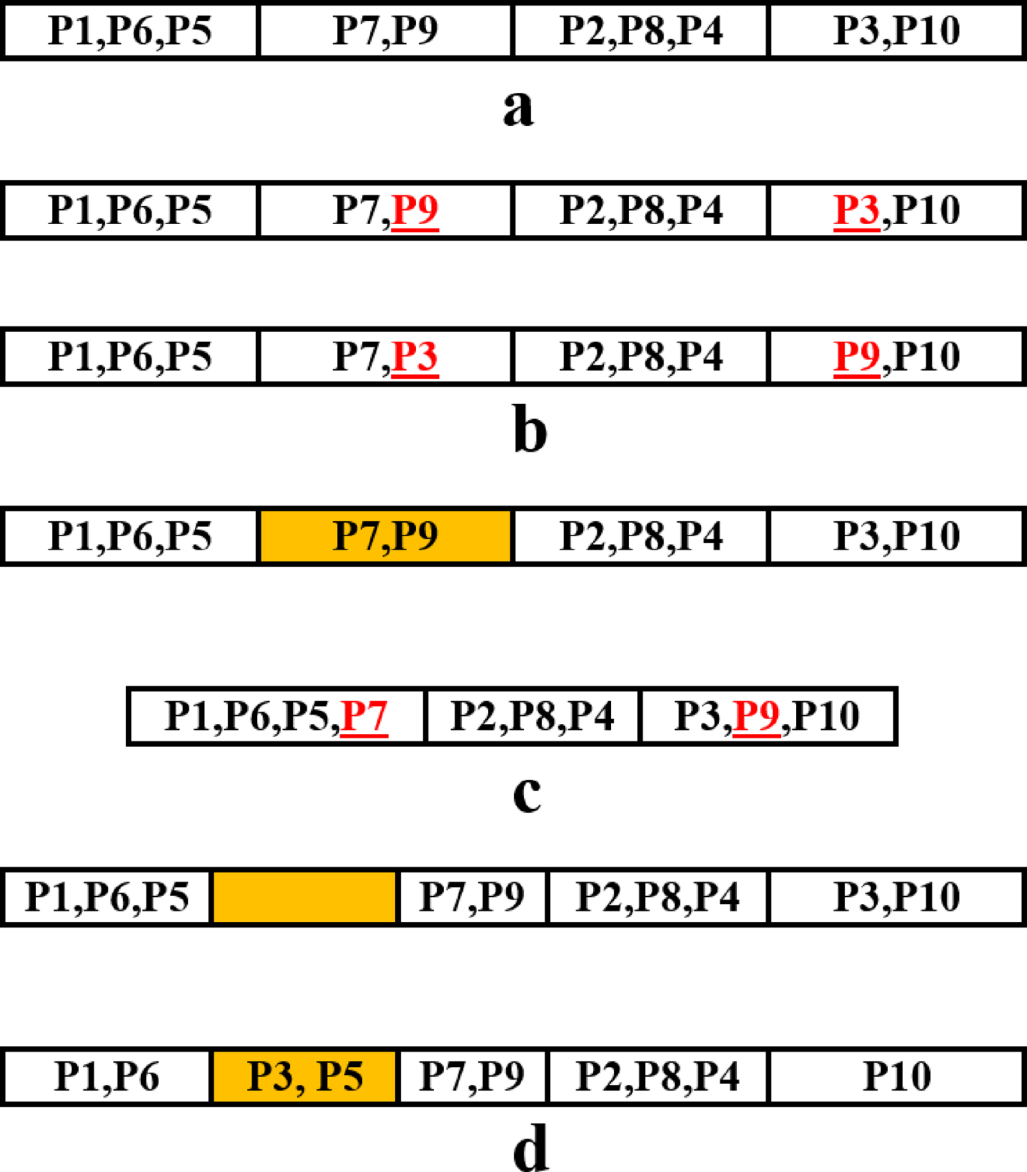
(**a**) A typical chromosome, (**b**) Mutation with replacing punches stations, (**c**) Mutation with deleting a station, (**d**) Mutation with adding a station.

In the present research, three methods are used for mutation. To illustrate these three methods, an example is used. Figure 6a shows a typical chromosome that is utilized for the mutation process. These methods are as follows:

- Two punches are randomly selected and their stations are replaced (Fig. 6b).
- One station is randomly deleted and its punches are randomly assigned to other stations (Fig. 6c).
- One station is added randomly between existing stations and some punches are randomly assigned to this station (Fig. 6d).

However, in each of these methods, the constraints between punches should be considered.

## Examples

To evaluate the performance of the proposed algorithm a prototype software is programmed in C# language in the Solidworks environment. The software receives the punch shapes, constraints between punches and GGA parameters as inputs and produces the distribution of punches in different stations as output.

**Fig. 7:**
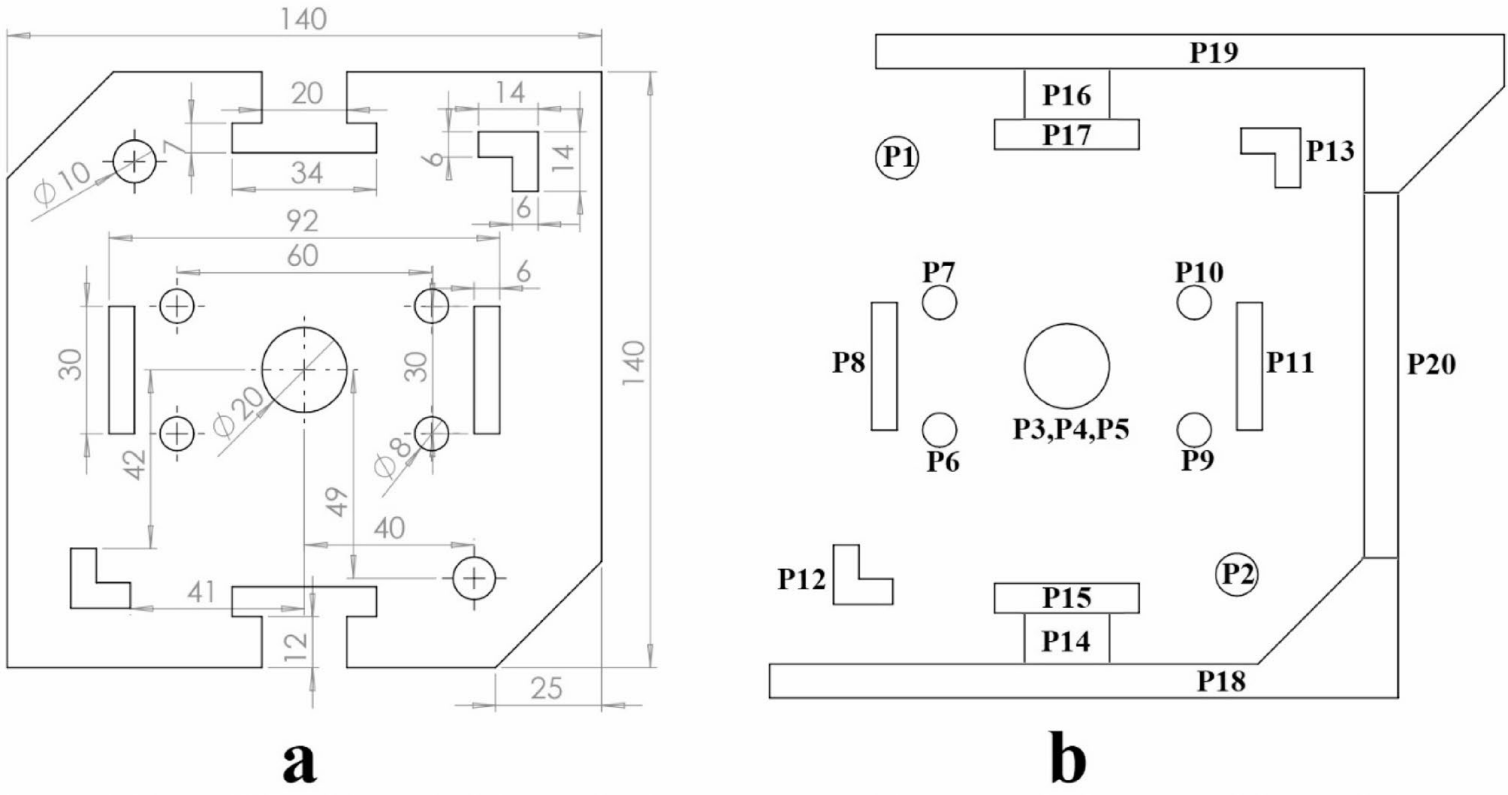
Example 1. (**a**) Sheet metal part, (**b**) Operating punches.

Four examples are provided in this section to demonstrate the algorithm’s performance. The first example is shown in Fig. 7a. Here twenty punches are used to produce the workpiece as shown in Fig. 7b. Constraints between punches are shown in Table 3. Two holes produced by punches P1 and P2 are used for piloting. Therefore, they are assigned to the first station. Punch P20 is used for cutting off the workpiece. Therefore, it should be assigned to the last station. Three punches P3, P4 and P5 are used to produce the central hole and have priority constraints. The Punch P3 first pierces the hole, followed sequentially by P4 and P5 as shaving punches. Punches P6, and P7 are close to punch P8 so they have the intersection constraint with it. This is also applicable to punches P9, P10 and P11. Punch P15 should be operated after punch P14 and punch P14 should be operated after punch P18. This is true for punches P16, P17 and P19. Punch P20 is near punches P18 and P19. Therefore, punch P20 has the intersection constraint with them. The parameters of the GGA used in example 1 are as follows: population size = 40, number of generations = 50, crossover rate = 0.5, and mutation rate = 0.2.

**Table 3:** Constraints between punches for example 1.

Constraint	Punches with this constraint
Intersection	{P6,P8}, {P7,P8}, {P9,P11}, {P10,P11}, {P20,P18}, {P20, P19}
Priority	{P3,P4,P5}, {P18,P14,P15}, {P19,P16,P17}
Assignment in the First station	P1, P2
Assignment in the Last station	P20

**Fig. 8:**
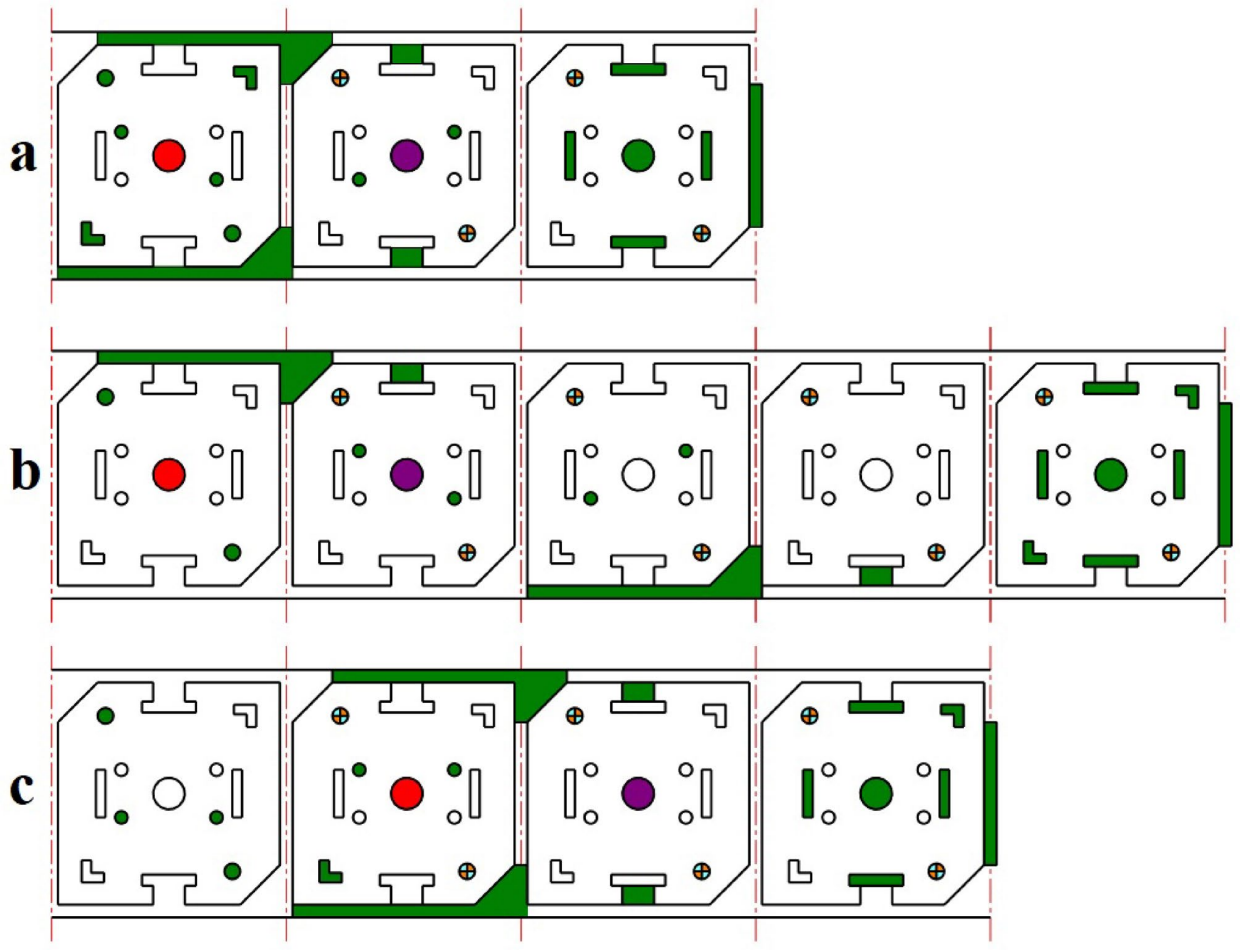
Strip layout design for example 1. (**a**) Proposed plan when the first objective is considered, (**b**) Proposed plan when the second objective is considered, (**c**) Proposed plan when two objectives are considered.

**Fig. 9:**
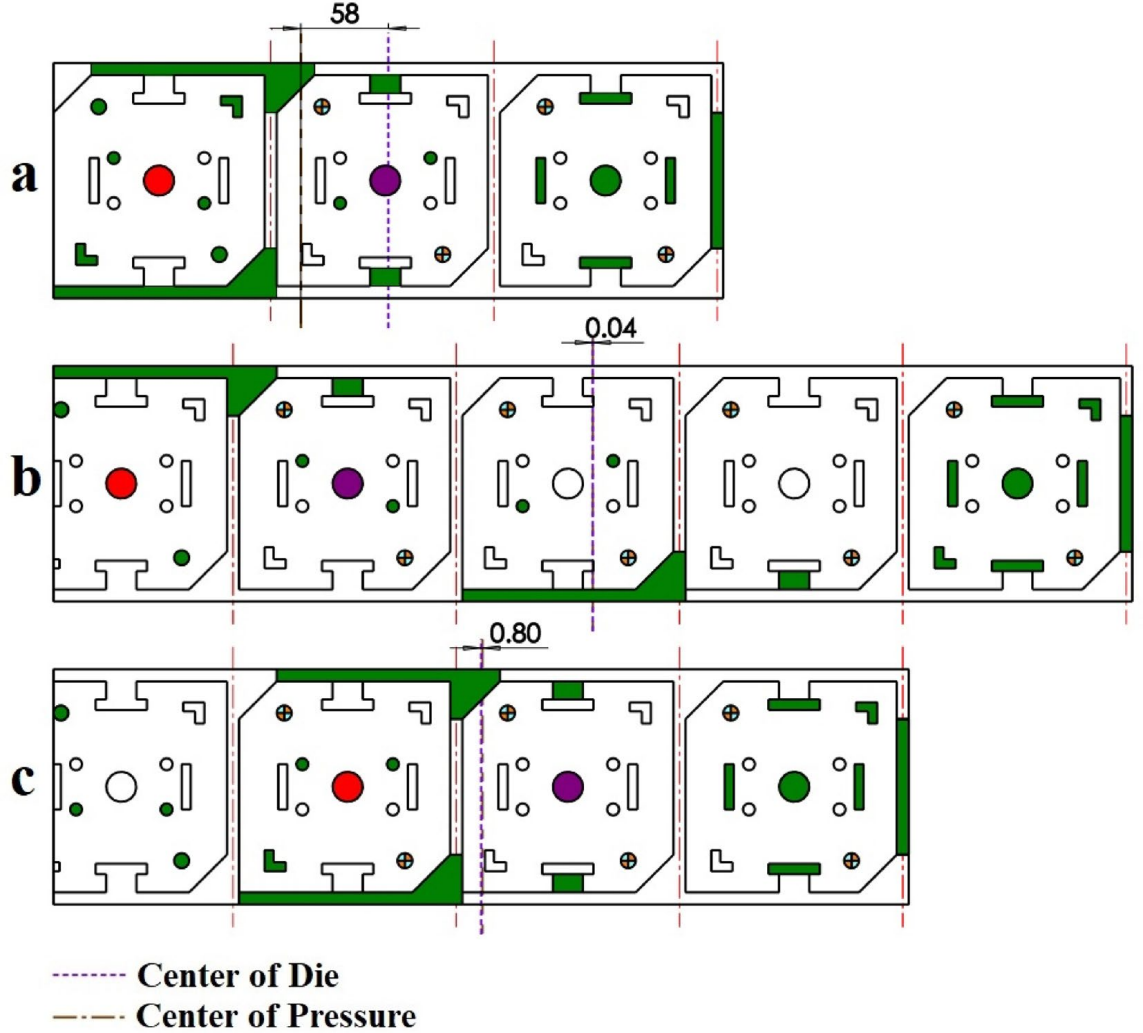
Distance between the die’s geometric center and pressure center for example 1. (**a**) Proposed plan when the first objective is considered, (**b**) When the second objective is considered, (**c**) When two objectives are considered.

The proposed strip layout design when the first objective is considered (minimum number of stations or *w*_*1*_ = 1 and *w*_*2*_ = 0) is shown in Fig. 8a, where three stations are proposed. When the second objective is considered (minimum torque or *w*_*1*_ = 0 and *w*_*2*_ = 1) is shown in Fig. 8b where five stations are proposed. Finally, when two objectives are equally weighted (minimum number of stations and minimum torque or *w*_*1*_ = 0.5 and *w*_*2*_ = 0.5) four stations are proposed (Fig. 8c). The distance between the Geometrical Die Center (GDC) and Pressure Center of the Die (PCD) for each of the proposed strip layout design is shown in Fig. 9. When the first objective is considered, the punches are not distributed properly so that distance between GDC and PCD is 58 mm (Fig. 9a). When the second objective is considered distribution of punch is very good. In this case, the distance is only 0.04 mm (Fig. 9b). When two objectives are considered the distribution of punches is also good where, the distance is 0.8 mm (Fig. 9c). As shown, when two objectives are considered (*w*_*1*_ = 0.5 and *w*_*2*_ = 0.5), although the number of stations has increased by one compared to the first state (*w*_*1*_ = 1 and *w*_*2*_ = 0), the distance between GDC and PCD is significantly decreased. Moreover, compared to the second state (*w*_*1*_ = 0 and *w*_*2*_ = 1), the distance between GDC and PCD is slightly increased, but the number of stations is reduced by one, which is desirable. The summary of results for example 1 is shown in Table 4.

**Table 4:** Summary of results for example 1.

State	Number of stations	GDC–PCD distance (mm)	Presence of idle stations
First state (*w*_*1*_ = 1 and *w*_*2*_ = 0)	3	58	No
Second state (*w*_*1*_ = 0 and *w*_*2*_ = 1)	5	0.04	No
Third state (*w*_*1*_ = 0.5 and *w*_*2*_ = 0.5)	4	0.8	No

**Fig. 10:**
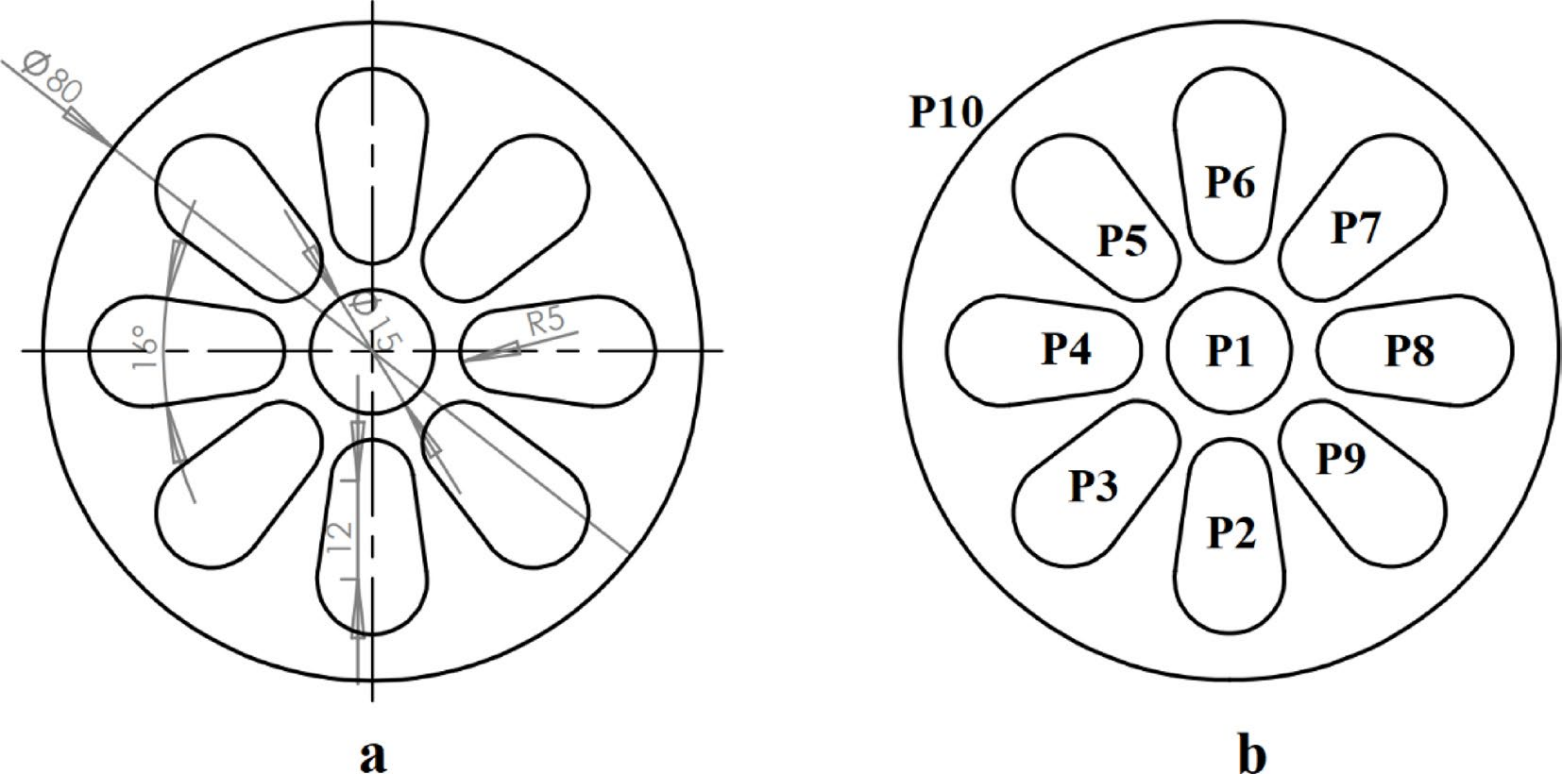
Example 2. (**a**) Sheet metal part, (**b**) Operating punches.

Figure 10 shows the second example part and its operating punches. As shown in Fig. 10b to produce this workpiece ten punches are needed. In this example, constraints between punches are shown in Table 5. A central hole produced by punch P1 is used for piloting. Therefore it is assigned to the first station. Punch P10 produces the external contour. Therefore, it should be assigned to the last station. This punch also has the intersection constraint with all other punches. Each of punches P2 to P9 is close to two punches on their sides and has the intersection constraint with them. Punch P1 also has the intersection constraint with punches P2 to P9. The parameters of the GGA used in example 2 are as follows: population size = 20, number of generations = 40, crossover rate = 0.5, and mutation rate = 0.2.

**Table 5:** Constraints between punches for example 2.

Constraint	Punches with this constraint
Intersection	{P1,P2}, {P1,P3}, {P1,P4}, {P1,P5}, {P1,P6}, {P1, P7}, {P1,P8}, {P1, P9}, {P10, P1}, {P10, P2}, {P10, P3},{P10,P4}, {P10,P5}, {P10,P6}, {P10,P7}, {P10,P8}, {P10,P9}, {P2,P3}, {P2, P9},{P3,P4}, {P4,P5}, {P5, P6},{P6, P7},{P7, P8},{P8, P9}
Priority	–
Assignment in the First station	P1
Assignment in the Last station	P10

**Fig. 11:**
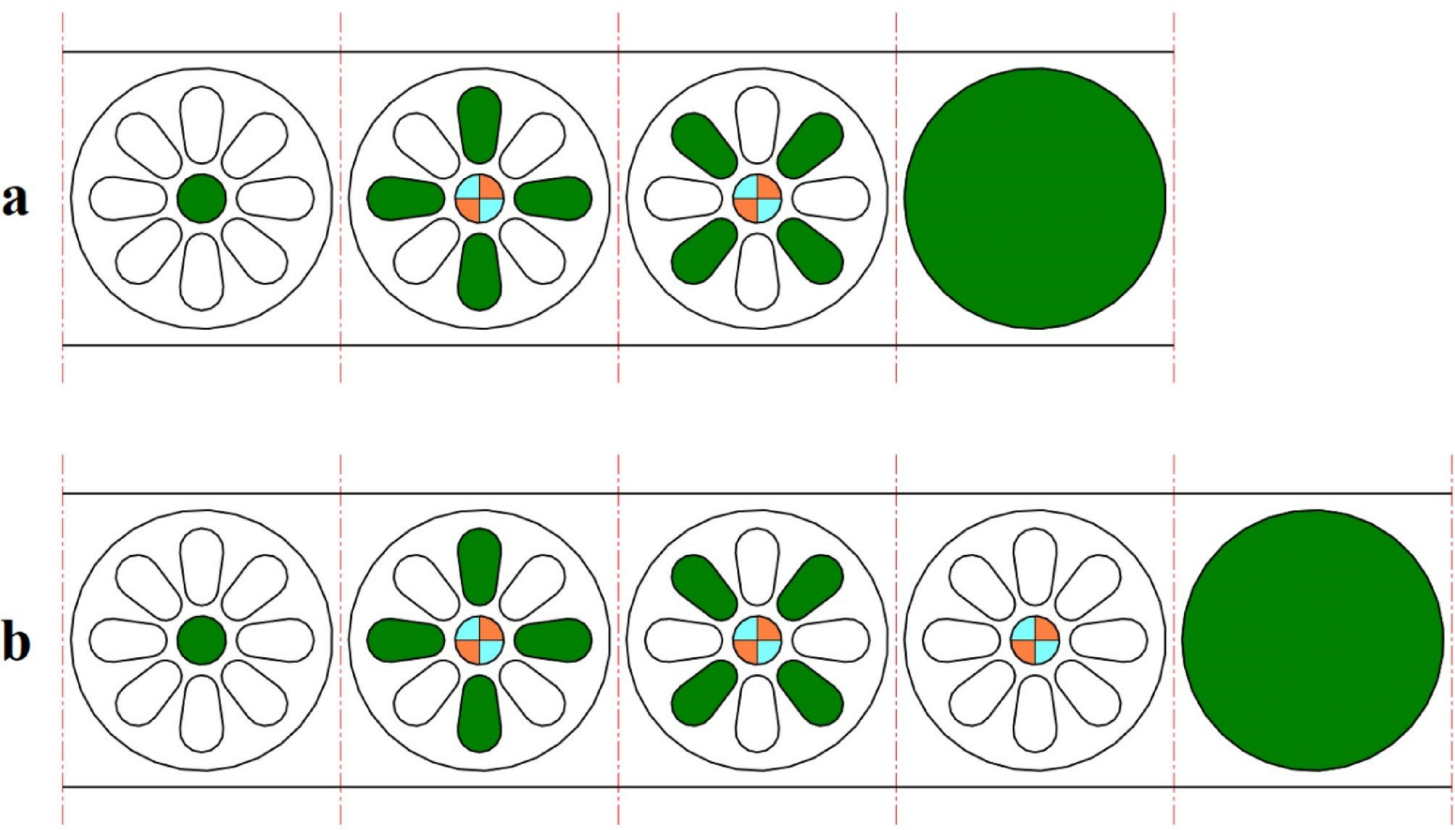
Strip layout design for example 2. (**a**) Proposed plan by Ghatrehnaby and Arezoo, (**b**) proposed plan by authors.

**Fig. 12:**
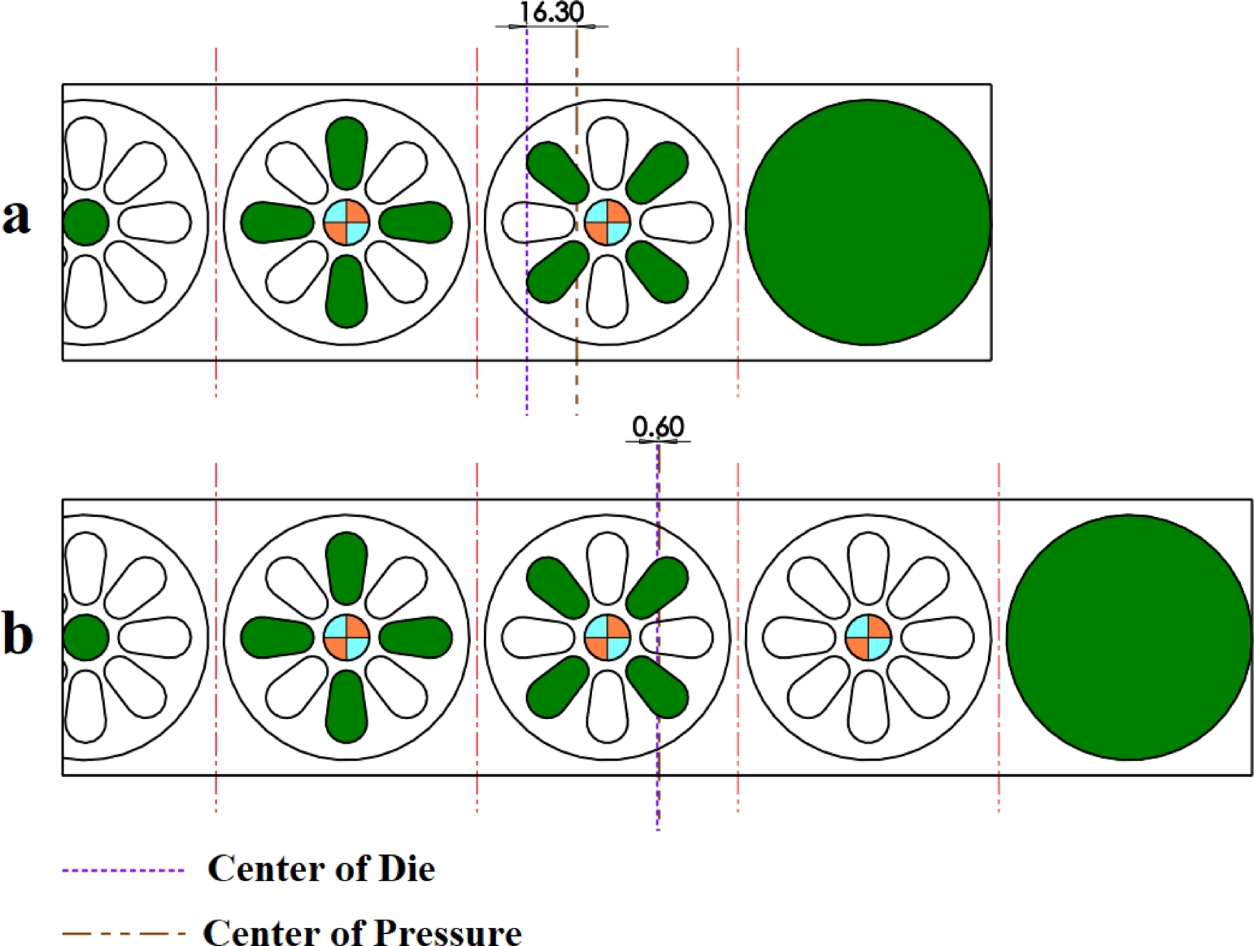
Distance between the die’s geometric center and pressure center for example 2. **(a)** Proposed plan by Ghatrehnaby and Arezoo, (**b)** Proposed plan by authors.

The proposed strip layout design by the Ghatrehnaby and Arezoo algorithm is shown in Fig. 11a. Also, the results of the proposed software when two objectives are considered are shown in Fig. 11b. As shown in Fig. 11 the Ghatrehnaby and Arezoo algorithm yields to a four station layout design while the proposed algorithm in the present work has suggested 5 stations. As shown in Fig. 11b the algorithm used in the present work suggests an idle station as the fourth station. The distance between GDC and PCD for each of the proposed strip layout designs is shown in Fig. 12. It is clear that although the number of stations proposed in the present work is higher but the distribution of punches in stations for the present work is more suitable regarding the distance between GDC and PCD. In fact, the proposed algorithm helped to a more optimal distribution of punches by introducing an idle station that effectively reduced the torque. The summary of results for example 2 is shown in Table 6.

**Table 6:** Summary of results for example 2.

Method	Number of stations	GDC–PCD distance (mm)	Presence of idle stations
Proposed plan by Ghatrehnaby and Arezoo	4	16.3	No
Proposed plan by authors (*w*_*1*_ = 0.5 and *w*_*2*_ = 0.5)	5	0.6	Yes

**Fig. 13:**
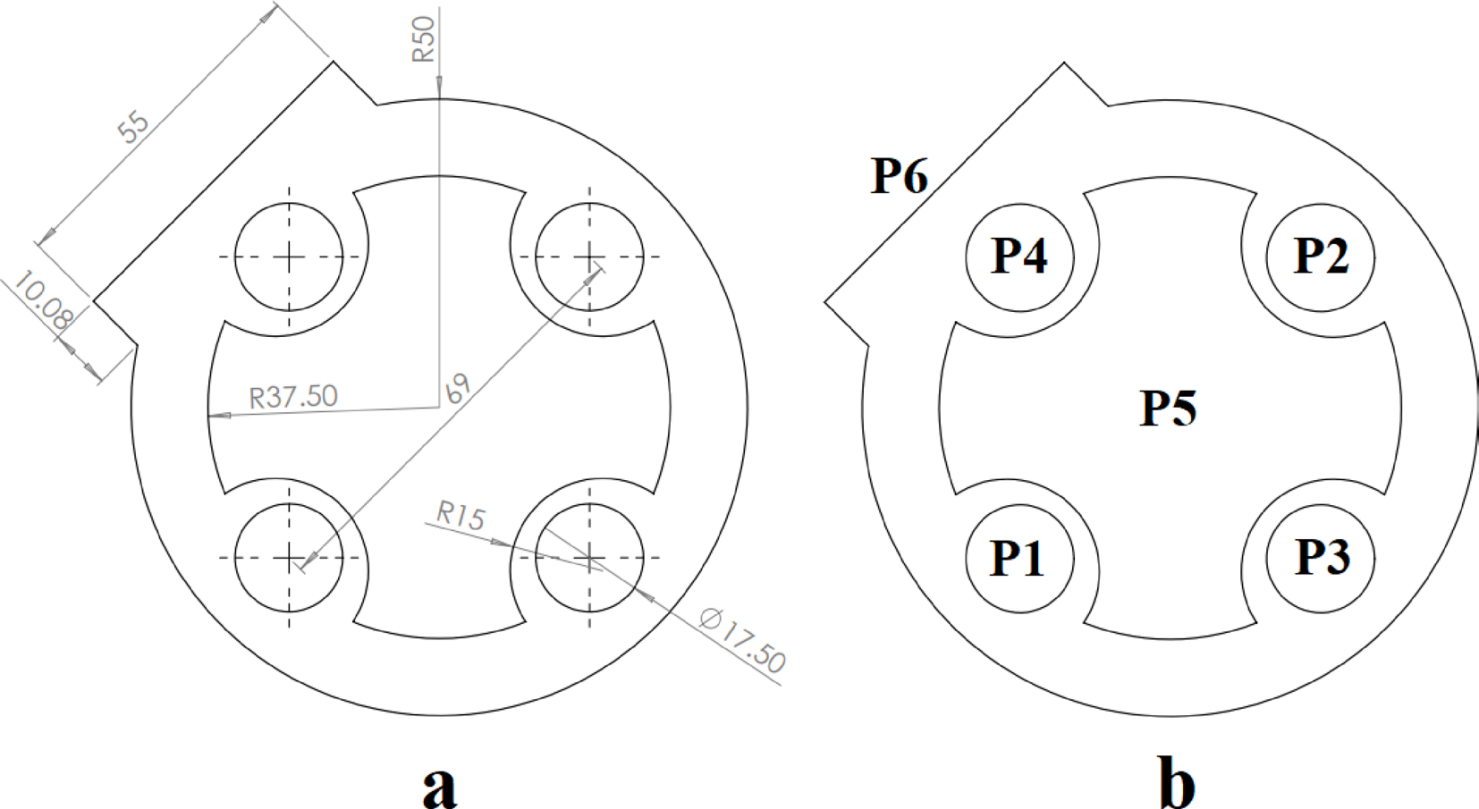
Example 3. (**a**) Sheet metal part, (**b**) Operating punches.

The third example which is taken from Moghaddam et al.^13^ and Dequan et al.^1^ is shown in Fig. 13. As shown in this Fig, six punches are needed to produce the workpiece. Constraints between punches for this example are shown in Table 7. Holes produced by punches P1 and P2 are used as piloting. Therefore these punches should operate at the first station. Punch P6 is used for cutting off the workpiece and is allocated to the last station. Also, this punch has the intersection constraint with all other punches. Punch P5 is too close to punches P1, P2, P3 and P4 so it has the intersection constraint with these punches. The parameters of the GGA used in example 3 are as follows: population size = 10, number of generations = 20, crossover rate = 0.5, and mutation rate = 0.2.

**Table 7:** Constraints between punches for example 3.

Constraint	Punches with this constraint
Intersection	{P1,P5}, {P2,P5}, {P3,P5}, {P4,P5}, {P1,P6}, {P2, P6}, {P3, P6}, {P4, P6}, {P5, P6}
Priority	–
Assignment in the First station	P1,P2
Assignment in the Last station	P6

**Fig. 14:**
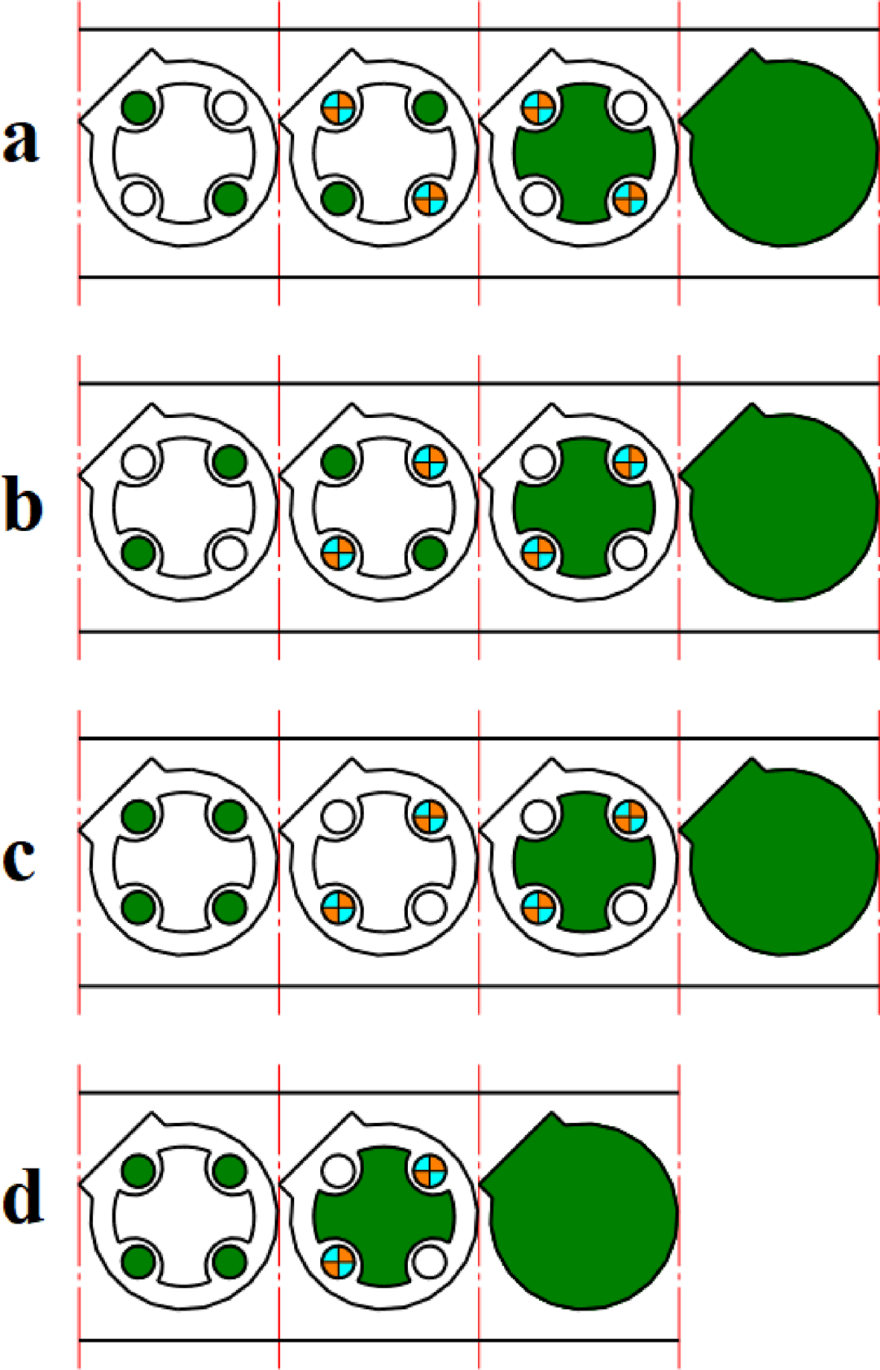
Strip layout design for example 3. (**a**) Proposed plan by Dequan et al., (**b**) Proposed plan by Moghaddam et al., (**c**) Modified plan by Moghaddam et al., (**d**) Proposed plan by authors.

The proposed strip layout design by Dequan et al., Moghaddam et al., and the present work is shown in Fig. 14a and d respectively. The distance between GDC and PCD for each of the designs is shown in Fig. 18. The algorithm proposed by Dequan et al. and Moghaddam et al. suggests four stations according to Fig. 14a and b. After proposing the layout design shown in Fig. 14b, Moghadam et al. modified it according to Fig. 14c and attempted to improve the torque equilibrium of the die by adding an idle station. The algorithm developed in the present work suggests only three stations.

**Fig. 15:**
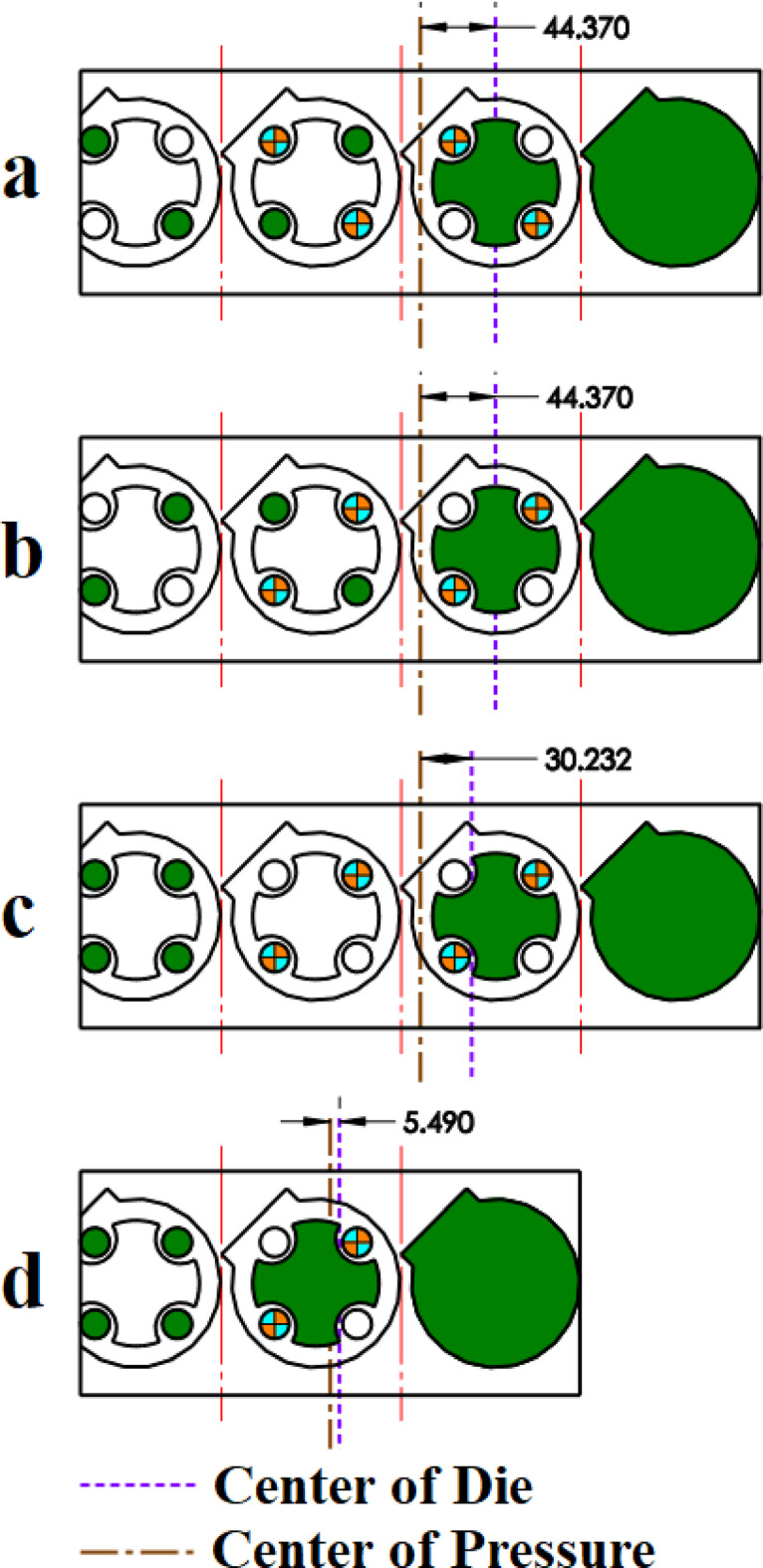
Distance between the die’s geometric center and pressure center for example 3. (**a**) Proposed plan by Dequan et al., (**b**) Proposed plan by Moghaddam et al., (**c**) Modified plan by Moghaddam et al., (**d**) Proposed plan by authors.

The proposed layouts are different in terms of torque equilibrium **(**Fig. 15**)**. The layouts for Dequan et al. and Moghaddam et al. are weak in terms of the distance between GDC and PCD. **(**Fig. 15a and b**)**. The distance value for these two proposed layouts is 44.37 mm. The modified layout by Moghadam et al., although improving the torque equilibrium compared to the initial design, still does not exhibit fully satisfactory performance in this regard** (**Fig. 15c**)**,so the distance between GDC and PCD is 30.23 mm. In the present work although fewer stations are suggested, also smaller deviation between GDC and PCD exists. The distance between GDC and PCD is 4.59 mm. **(**Fig. 15d**)**. The summary of results for example 3 is shown in Table 8.

**Table 8:** Summary of results for example 3.

Method	Number of stations	GDC–PCD distance (mm)	Presence of idle stations
Proposed plan by Dequan et al.	4	44.37	No
Proposed plan by Moghaddam et al.	4	44.37	No
Modified plan by Moghaddam et al.	4	30.23	Yes
Proposed plan by authors (*w*_*1*_ = 0.5 and *w*_*2*_ = 0.5)	3	5.49	No

**Fig. 16:**
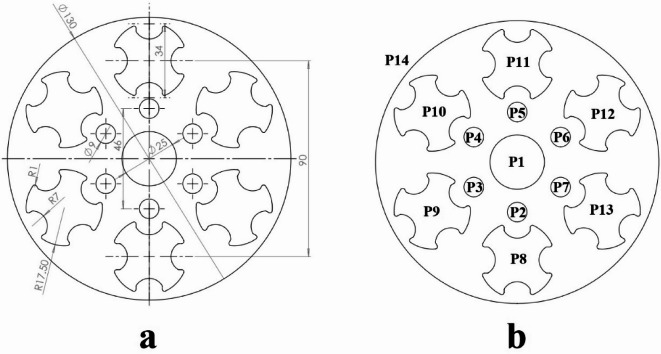
Example 4. (**a**) Sheet metal part, (**b**) Operating punches.

Figure 16 illustrates the fourth example part along with its operating punches. As depicted in Fig. 16b, producing this workpiece requires 14 punches. In this example, the constraints between the punches are detailed in Table 9.

The central hole, created by punch P1, is used for piloting and is therefore assigned to the first station. Punch P14, which produces the external contour, must be placed in the last station. This punch also has the intersection constraint with all other punches. Each of the punches P8 to P13 is positioned close to two adjacent punches and has the intersection constraint with them. Additionally, punch P1 has the intersection constraint with punches P2 to P7. Punches P2 and P8 are positioned close to each other. To prevent the deformation of hole 2, punch P8 should operate first. Therefore, there is a priority constraint between these two punches. The same constraint also exists between punches P3 and P9, P4 and P10, P5 and P11, P6 and P12, P7 and P13. The parameters of the GGA used in example 4 are as follows: population size = 30, number of generations = 40, crossover rate = 0.5, and mutation rate = 0.2.

**Table 9:** Constraints between punches for example 4.

Constraint	Punches with this constraint
Intersection	{P1,P2}, {P1,P3}, {P1,P4}, {P1,P5}, {P1,P6}, {P1, P7}, {P8, P9}, {P9, P10}, {P10, P11}, {P11, P12}, {P12, P13}, {P13, P8}, {P14, P1}, {P14, P2}, {P14, P3}, {P14, P4}, {P14, P5}, {P14, P6}, {P14, P7}, {P14, P8}, {P14, P9}, {P14, P10}, {P14, P11}, {P14, P12}, {P14, P13}
Priority	{P8,P2}, {P9,P3}, {P10,P4}, {P11,P5}, {P12,P6}, {P13, P7}
Assignment in the First station	P1
Assignment in the Last station	P14

**Fig. 17:**
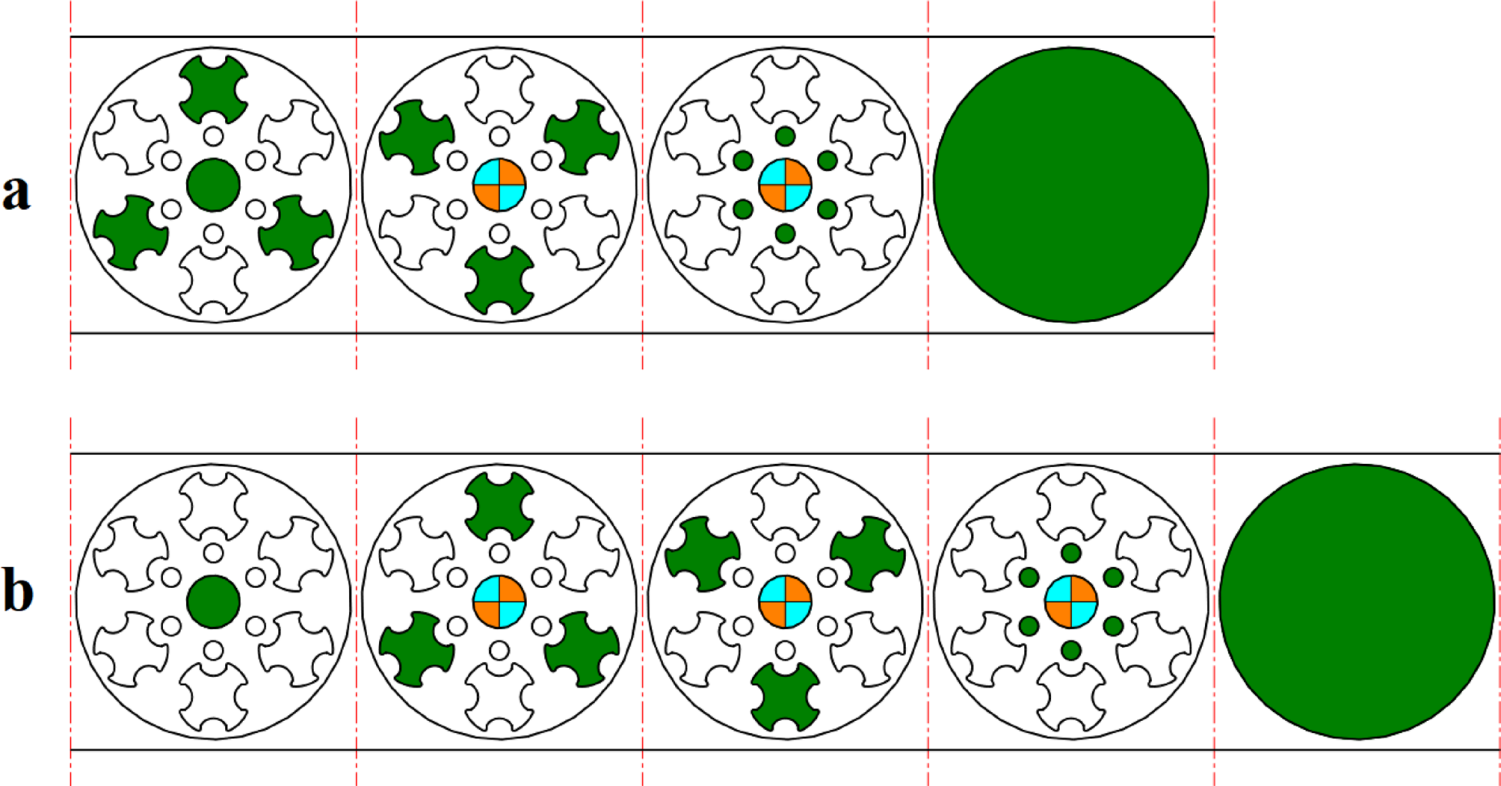
Strip layout design for example 4. (**a**) Proposed plan by Ghatrehnaby and Arezoo, (**b**) proposed plan by authors.

**Fig. 18:**
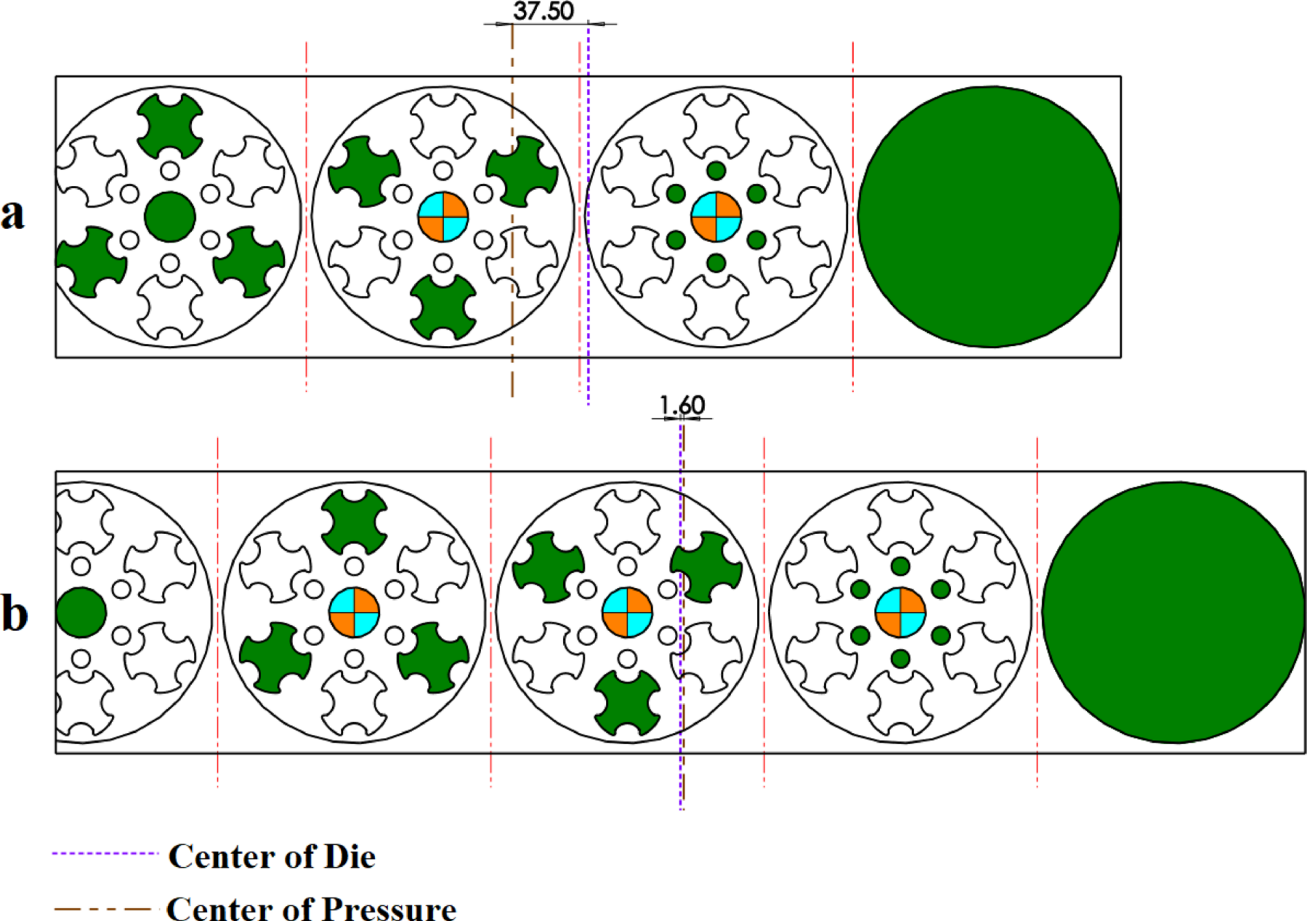
Distance between the die’s geometric center and pressure center for example 4. (**a**) Proposed plan by Ghatrehnaby and Arezoo, (**b**) Proposed plan by authors.

The strip layout design proposed by the Ghatrehnaby and Arezoo algorithm is shown in Fig. 17a, while the results of the proposed algorithm are shown in Fig. 17b. As seen in Fig. 17, the Ghatrehnaby and Arezoo algorithm results in a four-station layout design, whereas the algorithm proposed in the present work suggests a five-station layout. As shown in Fig. 18, although the proposed layout design by the current work suggests an additional station, the torque of the die has become significantly more balanced. The distance between GDC and PCD for the strip layout design proposed by the Ghatrehnaby and Arezoo algorithm is 37.5 mm while for author’s proposed algorithm is only 1.6 mm. As shown in Fig. 17b, the additional station is not idle. Therefore, the proposed algorithm is capable of balancing the die torque by adding stations (either active or idle) as needed. The summary of results for example 4 is shown in Table 10.

**Table 10:** Summary of results for example 4.

Method	Number of stations	GDC–PCD distance (mm)	Presence of idle stations
Proposed plan by Ghatrehnaby and Arezoo	4	37.5	No
Proposed plan by authors (*w*_*1*_ = 0.5 and *w*_*2*_ = 0.5)	5	1.6	No

### Discussion of limitations

Although the proposed method shows improved performance for progressive die layout design, several limitations should be noted:

#### Scalability to larger or more complex layouts

The current algorithm is well-suited for standard layout design challenges commonly seen in progressive die applications. However, when dealing with significantly larger or more complex problems, its scalability may become a concern. In such cases, more advanced approaches like Parallel Genetic Algorithms^44,45^ and divide-and-conquer evolutionary strategies^46^ could offer improved performance and efficiency.

#### Sensitivity to GGA parameters

Like many metaheuristic algorithms, the GGA can be influenced by its parameter settings, such as population size, the number of generations, or mutation rate. Initial tests involving moderate variation in these parameters revealed minimal impact on the final outcomes.

#### Conflicting constraints

The algorithm is capable of handling a variety of constraints, provided they are logically consistent. However, in cases where constraints directly conflict for instance, if one rule requires a punch to operate before another, while a separate rule demands the opposite the algorithm cannot produce a valid solution. Ensuring constraint consistency is therefore essential before execution.

## Conclusion

In this paper, a new approach for automatic strip layout design of progressive dies is proposed. Initially, using the weighted metric method and considering two objectives of minimum number of stations and minimum torque, a two-objective function for solving the problem is proposed. Then the different constraints that can exist between the punches are expressed. Considering the constraints between the punches and using the Grouping Genetic Algorithm the problem is optimized. A prototype software based on this new approach is developed. This software is tested with some examples. The results are compared with the strip layout designs proposed by other researchers, and the comparison demonstrates that the proposed algorithm achieves improved performance, since it simultaneously considers both objectives and if necessary, adds an idle or active station to improve the torque balance.

## Data Availability

All data generated or analysed during this study are included in this published article.

## References

[1] Dequan, Y. et al. Research of knowledge-based system for Stamping process planning. *Int. J. Adv. Manuf. Technol.* **29**, 663–669 (2006).

[2] Kumar, S. & Singh, R. A short note on an intelligent system for selection of materials for progressive die components. *J. Mater. Process. Technol.* **182** (1–3), 456–461 (2007).

[3] Kumar, S. & Singh, R. An intelligent system for automatic modeling of progressive die. *J. Mater. Process. Technol.* **194** (1–3), 176–183 (2007).

[4] Kumar, S. & Singh, R. Automation of strip-layout design for sheet metal work on progressive die. *J. Mater. Process. Technol.* **195** (1–3), 94–100 (2008).

[5] Kumar, S. & Singh, R. An expert system for selection of piloting for sheet metal work on progressive die. (2008).

[6] Ghatrehnaby, M. & Arezoo, B. A fully automated nesting and piloting system for progressive dies. *J. Mater. Process. Technol.* **209** (1), 525–535 (2009).

[7] Ghatrehnaby, M. & Arezoo, B. New mathematical approach for automatic piloting in computer aided progressive die design. *Proceedings of the Institution of Mechanical Engineers, Part B: Journal of Engineering Manufacture* **224**(12), 1879–1893 (2010).

[8] Ghatrehnaby, M. & Arezoo, B. Automatic piloting in progressive dies using medial axis transform. *Appl. Math. Model.* **34** (10), 2981–2997 (2010).

[9] Ghatrehnaby, M. & Arezoo, B. Automatic strip layout design in progressive dies. *J. Intell. Manuf.* **23**, 661–677 (2012).

[10] Afshari, M. & Arezoo, B. Development of an integer linear programming for automatic layout design in blanking and piercing progressive dies. *Iran. J. Manuf. Eng.* **9** (8), 12–25 (2022).

[11] Jia, Z. X. et al. Computer-aided structural design of punches and dies for progressive die based on functional component. *Int. J. Adv. Manuf. Technol.* **54**, 837–852 (2011).

[12] Lin, B. T. et al. Development of an automated structural design system for progressive dies. *Int. J. Adv. Manuf. Technol.* **68**, 1887–1899 (2013).

[13] Moghaddam, M., Soleymani, M. & Farsi, M. Sequence planning for Stamping operations in progressive dies. *J. Intell. Manuf.* **26** (2), 347–357 (2015).

[14] Moghaddam, M., Farsi, M. & Anoushe, M. Development of a new method to automatic nesting and piloting system design for progressive die. *Int. J. Adv. Manuf. Technol.* **77**, 1557–1569 (2015).

[15] Zhang, J. & Spath, D. Progressive die cost Estimation based on lamination design and production scenario in the electric traction motor application. *Procedia Manuf.* **39**, 635–644 (2019).

[16] Li, G. et al. Accurate trimming line optimization of multi-station progressive die for complex automotive structural parts. *Int. J. Adv. Manuf. Technol.* **95**, 1185–1203 (2018).

[17] Arora, A. et al. Design & analysis of progressive die using SOLIDWORKS. *Mater. Today: Proc.* **51**, 956–960 (2022).

[18] Aly, S. et al. Optimization of strip-layout using graph-theoretic methodology for Stamping operations on progressive die: a case study. *Int. J. Simul. Multi. Design Optim.* **12**, 5 (2021).

[19] Shakkarwal, P., Kumar, R. & Sindhwani, R. Progressive die design and development using AutoCAD, in Advances in Engineering Design: Select Proceedings of FLAME 2020. Springer. pp. 531–539. (2021).

[20] Yang, Y. & Hinduja, S. Sequence planning of sheet metal parts manufactured using progressive dies. *Int. J. Adv. Manuf. Technol.* **124** (7), 2199–2214 (2023).

[21] Murena, E. et al. Development and performance evaluation of a web-based feature extraction and recognition system for sheet metal bending process planning operations. *Int. J. Comput. Integr. Manuf.* **34** (6), 598–620 (2021).

[22] Yang, Y. et al. Recognition of features in sheet metal parts manufactured using progressive dies. *Comput. Aided Des.* **134**, 102991 (2021).

[23] Skampardonis, N., Tsirkas, S. & Grammatikopoulos S. Design and analysis of an industrial, progressive die for cutting and forming. 2021.

[24] Wei, Y. et al. Robust methodology of automatic design for automobile panel drawing die based on multilevel modeling strategy. *Int. J. Adv. Manuf. Technol.* **91**, 4203–4217 (2017).

[25] Faraz, Z. et al. Sheet-metal Bend sequence planning subjected to process and material variations. *Int. J. Adv. Manuf. Technol.* **88** (1), 815–826 (2017).

[26] Salem, A. A. et al. Towards an efficient process planning of the V-bending process: an enhanced automated feature recognition system. *Int. J. Adv. Manuf. Technol.* **91**, 4163–4181 (2017).

[27] Raj Prasanth, D. & Shunmugam, M. Geometry-based Bend feasibility matrix for Bend sequence planning of sheet metal parts. *Int. J. Comput. Integr. Manuf.* **33** (5), 515–530 (2020).

[28] Sen, Y. et al. A research on bending process planning based on improved particle swarm optimization. in Intelligent Robotics and Applications: 14th International Conference, ICIRA Yantai, China, October 22–25, 2021, Proceedings, Part I 14. 2021. Springer. (2021).

[29] Jemal, A., Salau, A. O. & Wondimu, A. Finite element method–based multi-objective optimization of press-brake bending of sheet metal. *Int. J. Adv. Manuf. Technol.* **130** (9), 4263–4275 (2024).

[30] Ai, Z. et al. Classification-based process parameter recommendation in sheet metal forming. *J. Industrial Inform. Integr.* **34**, 100458 (2023).

[31] Ghaffarishahri, S. & Rivest, L. A prototype of an automated feature recognition algorithm for aerospace sheet metal parts. *Comput.-Aided Des. Appl.* **19** (4), 624–661 (2022).

[32] Kong, C. et al. Research on automatic analysis and layout system of the Stamping process for automotive panels and its key technologies. *Int. J. Adv. Manuf. Technol.* **129** (9), 4101–4120 (2023).

[33] Kong, C., Lei, J. & Zhou, X. Research on the intelligent design system for automotive panel die based on geometry and knowledge driven. *Int. J. Adv. Manuf. Technol.* **133** (1), 765–790 (2024).

[34] Xu, F. et al. Prediction of bending parameters and automated operation planning for sheet-metal bending orientated to graphical programming. *Int. J. Adv. Manuf. Technol.* **126** (5), 2191–2204 (2023).

[35] Li, R. et al. Recognition and pose Estimation method for stacked sheet metal parts. *Appl. Sci.* **13** (7), 4212 (2023).

[36] Fei, L. et al. Efficient process planning algorithm for complex sheet metal bending. *Comput. Integr. Manuf. Syst.* **29** (10), 3331 (2023).

[37] Rathod, V., Jha, P. & Sawai, N. Optical CAD modelling and designing of compound die using the python scripting Language. *Int. J. Interact. Des. Manuf. (IJIDeM)*. **17** (2), 981–991 (2023).

[38] Jundi, A. & Alaiwi, Y. Design and analysis of compound die to produce L-Shape product with 3 holes. *Math. Modelling Eng. Probl.* **11**(5), 1245 (2024).

[39] Ma, L. & Meng, F. Anomaly detection in the production process of Stamping progressive dies using the Shape-and Size-Adaptive descriptors. *Sensors* **23** (21), 8904 (2023). 37960602 10.3390/s23218904 PMC10650308

[40] Stefanovska, E. & Pepelnjak, T. Optimising predictive accuracy in sheet metal Stamping with advanced machine learning: A LightGBM and neural network ensemble approach. *Adv. Eng. Inform.* **65**, 103103 (2025).

[41] Molitor, D. A. et al. Identifying productivity-limiting factors in progressive die stamping: data-driven methodology for process optimization. *Prod. Eng. Res. Devel.* **19**, 575–587 (2025).

[42] Miettinen, K. *Nonlinear multiobjective optimization* Vol. 12 (Springer Science & Business Media, 1999).

[43] Falkenauer, E. *Genetic algorithms and grouping problems* (John Wiley & Sons, Inc., 1998).

[44] Harada, T. & Alba, E. Parallel genetic algorithms: a useful survey. *ACM Comput. Surv. (CSUR)*. **53** (4), 1–39 (2020).

[45] Ferigo, A. & Iacca, G. A GPU-enabled compact genetic algorithm for very large-scale optimization problems. *Mathematics* **8** (5), 758 (2020).

[46] Yang, P., Tang, K. & Yao, X. A parallel divide-and-conquer-based evolutionary algorithm for large-scale optimization. *IEEE Access.* **7**, 163105–163118 (2019).

